# A survey of surgical team members’ perceptions of near misses and attitudes towards Time Out protocols

**DOI:** 10.1186/1471-2482-13-46

**Published:** 2013-10-09

**Authors:** Arvid Steinar Haugen, Shamini Murugesh, Rune Haaverstad, Geir Egil Eide, Eirik Søfteland

**Affiliations:** 1 Department of Anaesthesia and Intensive Care, Haukeland University Hospital, Bergen, Jonas Liesvei 65, N-5021, Bergen, Norway; 2 Department of Clinical Science, Faculty of Medicine and Dentistry, University of Bergen, Bergen, Norway; 3 Section of Cardiothoracic Surgery, Department of Heart Disease, Haukeland University Hospital, Bergen, Norway; 4 Center for Clinical Research, Haukeland University Hospital, Bergen, Norway; 5 Department of Global Public Health and Primary Care, Faculty of Medicine and Dentistry, University of Bergen, Bergen, Norway

**Keywords:** Surgery, Operating room, Near misses, Medical errors, Checklist

## Abstract

**Background:**

Medical errors are inherently of concern in modern health care. Although surgical errors as incorrect surgery (e.g., wrong patient, wrong site, or wrong procedure) are infrequent, they are devastating events to experience. To gain insight about incidents that could lead to incorrect surgery, we surveyed how surgical team members perceive near misses and their attitudes towards the use of Time Out protocols in the operating room. We hypothesised that perceptions of near-miss experiences and attitudes towards Time Out protocols vary widely among surgical team members.

**Methods:**

This cross-sectional study (N = 427) included surgeons, anaesthetists, nurse anaesthetists, and operating room nurses. The questionnaire consisted of 14 items, 11 of which had dichotomous responses (0 = no; 1 = yes) and 3 of which had responses on an ordinal scale (never = 0; sometimes = 1; often = 2; always = 3). Items reflected team members’ experience of near misses or mistakes; their strategies for verifying the correct patient, site, and procedure; questions about whether they believed that these mistakes could be avoided using the Time Out protocol; and how they would accept the implementation of the protocol in the operating room.

**Results:**

In the operating room, 38% of respondents had experienced uncertainty of patient identity, 81% had experienced uncertainty of the surgical site or side, and 60% had prepared for the wrong procedure. Sixty-three per cent agreed that verifying the correct patient, site, and procedure should be a team responsibility. Thus, only nurse anaesthetists routinely performed identity checks prior to surgery (*P* ≤ 0.001). Of the surgical team members, 91% supported implementation of a Time Out protocol in their operating rooms.

**Conclusion:**

The majority of our surgical personnel experienced near misses with regard to correct patient identity, surgical site, or procedure. Routines for ensuring the correct patient, site, and surgical procedure must involve all surgical team members. We find that the near-miss experiences are a wake-up call for systematic risk reducing efforts and the use of checklists in surgery.

## Background

Medical errors are inherently of great concern in modern health care. Approximately 1-in-10 hospital in-patients experience an adverse event, and nearly two-thirds of these are associated with a surgical provider [1]. Adverse events in surgical patients are estimated to be highly preventable in 48% of the cases [2]. Although incorrect surgery—defined as wrong patient, wrong site, or wrong procedure—occurs infrequently, surgical teams recognise these as devastating events to experience. A case review of wrong-site craniotomies identified five major contributing factors: communication breakdowns, inadequate preoperative checks, technical factors and imaging misidentifications, and simple human errors [3]. A near-miss event study of orthopaedic procedures and noncompliance to antimicrobial prophylaxis identified causes as human, organisational, and material factors [4]. Systematic use of a checklist prior to incision as a preventive effort was recommended by 40% of the declarants, along with improved communication between anaesthetists and surgeons [4]. In a malpractice claim study in the Netherlands, wrong patient, wrong site, or wrong procedure were identified in 16% (46/294) of cases and were suggested to be preventable if the staff had followed the comprehensive Surgical Patient Safety System (SURPASS) of checklists [5]. Ensuring the correct patient, correct site, and correct procedure is vital in order to avoid incorrect surgery. The Joint Commission (JC), an independent organisation accrediting and certifying health care organisations in the United States, underlines the importance of implementing a range of risk reduction strategies to prevent wrong-site surgery. Amongst these recommendations is that the surgical team should take a time-out and use active communication techniques to verify they are dealing with the correct patient, site, and procedure [6].

Several studies show reductions in both mortality and morbidity after introducing surgical checklists [7-10]. Improved communication and shared responsibility within the health care teams may contribute to the elimination of wrong-site surgery [3,11,12]. As error management depends on human skills and reliability of surgical team members, a systematic approach is required [13].

In a concurrent safety climate study performed at our hospital (Haukeland University Hospital) prior to the introduction of surgical checklists, anaesthetic personnel scored significantly higher than operating room nurses and surgeons on frequency of near-miss events reported [14]. In general, the safety climate perceptions were significantly underscored when compared with hospital staff in the U.S., e.g. 31% to 62% on frequency of near-miss events reported [14]. Report of near-miss perceptions is considered to have several advantages as fewer barriers, limited liability and patterns which could be captured and used to improve surgical care [15,16]. To better understand the nature of near misses in surgery, a deeper understanding of surgical team members’ perceptions and attitudes has been warranted.

We investigated surgical team members’ perceptions of incorrect surgery and how the correct patient, correct site, and correct procedure were ensured in daily routines. We hypothesised that perceptions of near-miss experiences and attitudes towards Time Out protocols vary widely amongst surgical team members.

## Methods

We surveyed surgical team members’ perceptions and attitudes using a cross-sectional design that included 427 surgical team members (surgeons, operating room nurses, anaesthetists, and nurse anaesthetists). Participants were identified from employment lists provided by staff managers. All participants worked in the central operating unit (COU).

### Organisational context

The study was conducted at a tertiary university hospital in western Norway (Haukeland University Hospital). The COU has 22 operating rooms, conducting 15,000 operations annually. The operations comprised orthopaedic; cardiothoracic; neuro; ear, nose, and throat (ENT); plastic; urologic; gastroenterological; and endocrine surgeries. The operating room personnel except for surgeons are administered by the Department of Anaesthesia and Intensive Care. Time Out protocols, safety climate instruments, and root cause analysis were not conducted either before or when this study was carried out in February 2009. One section of the care unit was familiar with a pre-anaesthetic induction checklist [17]. An electronic adverse event reporting system was used throughout the hospital. In a concurrent mapping of organisational (neurosurgery and orthopaedic) standards, we found that patient identity was routinely checked at the ward, and further name tags were scanned when patients entered the COU. However, if there was uncertainty about patient identity or body site markings, patients were not routinely returned to the ward (Personal communication by SM, ASH and ES).

### Questionnaire

The questionnaire consisted of 14 items, 11 of which had dichotomous responses (0 = no; 1 = yes) and 3 of which had responses scored on an ordinal scale (items 7–9) (never = 0; sometimes = 1; often = 2; always = 3). Items reflected team members’ own experience of near misses or mistakes; their strategies for ensuring the correct patient, side, and procedure; questions about whether they believed that such mistakes could have been avoided by using the Time Out protocol; and how they might accept the introduction of the protocol into the COU. The survey also contained an open-ended question that allowed respondents to offer their opinions on the topic. The 14 items are listed in Table 1. The survey also included questions about participant characteristics such as profession, experience, and gender.

**Table 1:** Time Out survey at Haukeland University Hospital, Bergen, Norway, 2009

	
1	Have you observed a wrong patient being brought into the operating room?
2	Have you experienced uncertainty about patient identity in the operating room?
3	Have you observed wrong positioning of patient prior to surgery?
4	Have you experienced uncertainty about operation side prior surgery?
5	Have you observed preparation for wrong procedure?
6	Is the responsibility for checking patient identity, operation side, and operation procedure a joint responsibility?
7	Do you check patient identity prior to each operation?
8	Do you verify the correct site/side prior to each operation?
9	Do you verify the correct surgical procedure prior each operation?
10	Do you believe incorrect surgery is performed as a result of not verifying patient identity, side, and procedure?
11	Does anyone use the Time Out protocol in your operating room?
12	Do you believe the Time Out protocol can prevent incorrect surgery? ^a^
13	Do you find the Time Out protocol useful?
14	Would you like to use a Time Out protocol in your operating room?

^a^Incorrect surgery: wrong patient, wrong side/site, or wrong procedure.

Information given to respondents prior to item 11: Time Out protocol is performed prior to skin incision. The team verifies correct patient identity, correct site, and correct procedure. The operation does not start until all agree. One dedicated person in the team coordinates the checklist. Time Out is documented in patient records.

The survey was pilot tested by 10 research fellows and medical staff who reviewed the functionality of the electronic system and the adequacy and relevance of the questions. The expert panel regarded the validity of the questionnaire as appropriate in terms of its design and content. The initial review resulted in minor adjustments of sample characteristics; i.e., changing the self-report of professional experiences into years practising instead of categorising professional experiences into different groups.

### Data collection

The survey was distributed to all eligible medical personnel through our hospital email system. We used a web-based questionnaire with an information letter and a direct link to the questionnaire itself. The questionnaire had to be submitted within four weeks. To increase the interest of potential respondents prior to distribution, we also promoted the survey on the hospital intranet page and on wall posters in the COU. The hospital managers provided access for survey recruitment at staff meetings.

### Statistics

Descriptive statistics were used to quantify the respondents’ characteristics. Analysis of differences within professions and between professions, as well as the safety items, was performed with the Pearson’s chi-squared test. *P*-values were two-sided, and the significance level was 0.05. All data were analysed using SPSS version 18 [18]. Qualitative content analysis was used to evaluate surgical team members’ experiences of incorrect surgery and to assess patient safety issues reported in the open-ended question [19].

### Ethics

The study was reviewed and approved by the Committee for Medical Research Ethics of the Western Norwegian Regional Health Authorities and the Norwegian Social Sciences Data Services. Permission to perform the study was also granted and endorsed by the hospital management. Respondents agreed to participate by answering the questionnaire. The Department of Research and Development preserved electronic data anonymity and assisted in administering the survey. Data were secured in the hospital research server; the study complied with the Helsinki Declaration [20].

## Results

**Figure 1:**
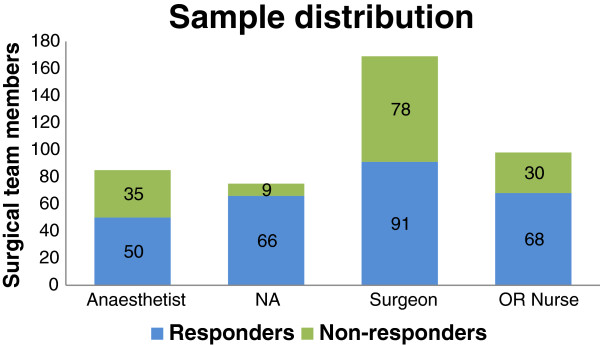
Sample distribution of responders and non-responders in the Time Out Survey at Haukeland University Hospital, Bergen, Norway, 2009.

**Figure 2:**
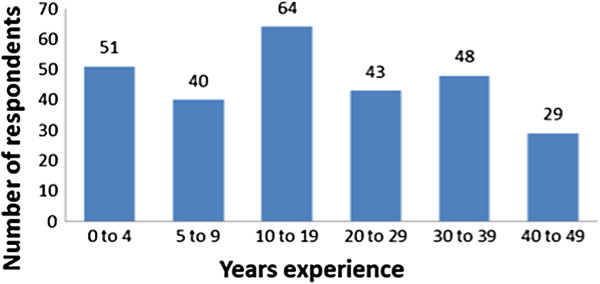
Sample characteristics of work experience at Haukeland University Hospital, Bergen, Norway, 2009.

Answers were received from 64% (275/427) of staff, including 54% (91/169) of the surgeons, 59% (50/85) of the anaesthetists, 69% (68/98) of the operating room nurses, and 88% (66/75) of the nurse anaesthetists (Figure 1). The numbers of responses from males and females were equal. There was a significant difference (*P* = 0.032) across genders on responses to the question about uncertainty of the correct procedure: Males were less uncertain. Agreement that the responsibility for checking patient identity, site, and procedure was a joint team task was more evident in females (*P* = 0.003). The mean (SD) years of experience was 19 (13.8) years. Figure 2 presents the characteristics of the respondents.

**Figure 3:**
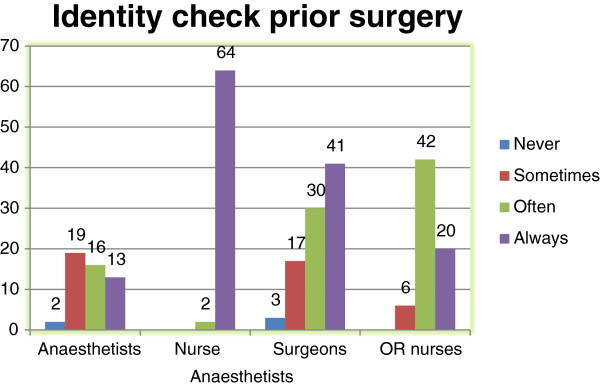
**Histograms showing how often patient identity is checked prior to each surgery by profession.** The Time Out Survey of surgical team members at Haukeland University Hospital, Bergen, Norway, 2009.

Thirty-eight per cent (105/275) of the participants observed instances of failure to confirm patient identity, and 19% (51/275) observed the wrong patient brought into the operating room. In addition, 43% (119/275) observed that patients were positioned on the wrong side before incision. A total of 81% (222/275) experienced instances in which the anatomical side or surgical site of patients was not confirmed, while 60% (164/275) experienced occasions in which the staff mistakenly prepared for a procedure different from the procedure actually planned. There was significant variability (*P* ≤ 0.001) amongst practitioners of different health care professions with regard to experiencing safety issues before surgery (Table 2). Figure 3 shows how members of different health care professions differed in performing patient identity checks prior to surgery. When asked whether the three original Universal Time Out protocol checkpoints (verifying correct patient, site, and procedure) should be a joint team responsibility, 63% (175/275) agreed, 16% (45/275) disagreed, and 20% (54/275) were uncertain about the responsibility. As many as 90% (247/275) of the participants believed that failure to verify the site or procedure prior to surgery is an important factor in causing surgical incidents. Only 5% (14/275) were familiar with the Universal Time Out Protocol. The majority of participants, in all 96% (263/275), believed that a Time Out protocol could aid in preventing wrong surgery, and 91% (250/275) agreed that a Time Out protocol should be implemented in our operating rooms.

**Table 2:** Surgical team members’ experiences with near misses in the operating room

	**Surgical team members’ profession**																				
**Experiences with:**	**Anaesthetist**				**NA**				**Surgeon**				**OR Nurse**				**Total**				
	**(n = 50)**				**(n = 66)**				**(n = 91)**				**(n = 68)**				**(N = 275)**				
	**Yes**		**No**		**Yes**		**No**		**Yes**		**No**		**Yes**		**No**		**Yes**		**No**		**P-**
	**n**	**(%)**	**n**	**(%)**	**n**	**(%)**	**n**	**(%)**	**n**	**(%)**	**n**	**(%)**	**n**	**(%)**	**n**	**(%)**	**n**	**(%)**	**n**	**(%)**	**value**^**a**^
**Wrong patient in OR**	14	(28)	36	(72)	13	(20)	53	(80)	11	(12)	80	(88)	13	(19)	55	(81)	51	(19)	224	(81)	0.136
**Uncertain of patient identity**	31	(62)	19	(38)	30	(46)	36	(54)	21	(23)	70	(77)	23	(34)	45	(66)	105	(38)	160	(62)	<0.001
**Prepared for another procedure**	26	(52)	24	(48)	42	(64)	24	(36)	49	(54)	42	(46)	47	(69)	21	(31)	154	(60)	111	(40)	<0.001
**Uncertain of correct site**	46	(92)	4	(8)	58	(88)	8	(12)	53	(58)	38	(42)	65	(96)	3	(4)	222	(81)	53	(19)	<0.001
**Wrong positioning of patient**	21	(42)	29	(58)	31	(47)	35	(53)	32	(35)	59	(65)	35	(52)	33	(48)	119	(43)	156	(57)	0.197

*Abbreviations: NA* Nurse anaesthetist, *OR* Operating room.

^a^P-value based on Pearson’s chi-squared test.

Surgical team members’ experiences with near misses in the operating room (wrong patient, uncertainty about correct patient and correct site, preparing for wrong procedure and positioning) at Haukeland University Hospital, Norway, 2009.

Forty-four participants answered the open-ended question and included their reflections on attitudes and perceptions of the Time Out protocol and safety issues. Of these comments, 14 were in favour of the Time Out protocol: For example, ‘… *this is a good idea. Hopefully it could be implemented at our COU; so go for it!*’ ‘*Good, I have been missing Time Outs for a long time*’*.*

The themes emerging from the content analysis were safety issues that involve ensuring patient identity and communication failures. A frequent problem was that the electronic surgical operating plan system was inaccurate, at times, regarding the planned procedure or site and the surgical or anaesthetic equipment required. Possible reasons for the inaccuracies were limitations in the electronic computer program or incomplete notes of variables on the request form. Another safety concern was that surgeons were unaware of patient identity and site of procedure when they arrived in the operating room. One respondent stated: ‘*I have been here for 15 years now and have experienced one or two actual wrong site surgeries in all*’*.*

## Discussion

Overall the results show a lack of organisational safety culture development. Only 63% of the respondents stated that verifying the correct patient, site, and procedure is a joint team responsibility. Routines for ensuring the correct patient, correct site, and correct surgical procedure were practised significant differently (P < 0.001) amongst our medical professionals (Table 2). Figure 3 presents the differences between these professionals in ensuring patient identity prior to every surgery. Nurse anaesthetists are the gatekeepers of correct patient identity in our COU, and thus are more responsible than other team members for verifying patient identity during transfer to the operating room. Hence, the other surgical team members seemed to rely on the locally established system about whether to accept or eventually reveal incorrect patient situations in the operating room. We regard this as an unsatisfactory safety assurance system. In our view, all involved medical personnel must check patient identity with regard to their own medical objectives. To address team responsibility for verifying the correct patient and identity we strongly recommend routinely use of a surgical safety checklist [7,10].

In the present study, survey comments of surgical team members underline that various flaws in the operation planning system or incorrect information ahead of surgery contributed to the extensive experience of miscommunication. This finding corresponds with a concurrent safety climate study in our hospital that found surgical patient information handover between departments or units to be poor [14]. Other studies underline the importance of communication [3,11,12]. Safety attitudes in an organisation should be monitored through safety climate questionnaires and interviews of staff as part of a systematic program aiming to improve safety.

### Wrong patient

Thirty-eight per cent of the participants experienced instances of unconfirmed patient identity in the operating room, and 19% experienced wrong patients being brought into the operating room. The number of surgical team members experiencing incorrect surgeries (i.e., wrong patient brought into the operating room, surgery performed on the wrong site, or the wrong procedure planned) seems high. According to our results, these experiences are expected to occur at least once during one’s professional career. In a study of wrong-site surgeries reported to the Pennsylvania Patient Safety Reporting System, Clarke et al*.* found that of 433,528 reports, 427 notifications were about wrong-site surgery. Of these, 70% were wrong side, 56% were near misses, 14% were wrong location/level, 9% were wrong procedure, and 8% were actually performed on the wrong patient [21].

### Wrong site

Experiences of surgery planned for the wrong site/side (81%) and wrong positioning of the patient on the operating table (43%) revealed that near misses were familiar to most of the surgical team members in our study. Incidents of near misses are prone to occur up to 300 times more often than actual events [15]. A literature review underlines the advantages of reporting near misses as they are more frequent than adverse events and with fewer barriers to reporting [16]. A study of wrong-site surgery reported that 16% (173/1050) of the surgeons were about to operate on the wrong site but were warned prior to incision, and 21% (217/1050) reported that they had performed wrong-site surgery at least once [22].

Our study did not examine the actual incidence of incorrect surgeries. However, from our results we could infer that wrong site/side near misses would probably mitigate by systematic site marking prior to surgery. Marking the site or side before surgery is one of the recommendations of the JC to prevent incorrect surgery [6]. This includes preoperative routines for when site marking should be performed, how the site should be marked, and by whom. Marking the wrong site or patient may occur in scenarios in which surgeons rush to the operating room to mark sites that failed to be marked in the preoperative ward. Multiple surgeons performing several procedures on the same patient and unusual time pressures are amongst the factors contributing to wrong-site surgery [11].

The near misses identified in the present study highlight the need for implementing systematic safety efforts in surgical care. Using checklists that cover the entire surgical pathway, from admission to discharge, as described in the SURPASS comprehensive checklist system, can further improve surgical care and prevent incorrect surgery [5].

### Wrong procedure

Of the surgical team respondents, 60% experienced planning the wrong procedure. The answers to the open-ended question of our survey revealed various reasons. A common reason was insufficient communication between the surgeons and the operating room nurses or anaesthetic staff due to lack of information in the electronic planning system or lack of information from the surgeon.

According to previous reports, performing a wrong procedure is not the most frequent adverse event [21]. However, our study suggests that preoperative planning and communication in our hospital can be improved. The National Patient Safety Agency reported that implementing the WHO safety surgical checklist could reduce incorrect surgeries [12,23]. Panesar et al. found that checklists could potentially mitigate 14.9% of near misses and 83.3% of harmful events [12,23]. In another study of surgical checklists, the procedure check during the Time Out was rated as ‘very important’ by nurses and ‘important to some degree’ by surgeons, indicating that different professionals perceive checklists differently [24].

### Attitudes towards protocols

Incorrect surgery occurs in the context of an organisation, teams, and culture. As 91% of the surgical team members in the present study had a positive attitude towards time-outs, the majority of professionals and groups welcomed a Time Out protocol in the operating room. Perceptions are prone to be influenced by the awareness evoked in the near-miss survey and by its results. Hence, these positive attitudes towards protocols seem to have paved the way for the subsequent, successful implementation of the WHO’s Surgical Safety Checklist in our hospital. Indeed, compliance to the three parts of the checklist was 77% to 85% [25]. Moreover, in the checklist-intervention group, there was a reduction in frequency of near misses reported, suggesting that the checklist had mitigated, to some degree, the problem of operating room mistakes [25].

An important finding of the present study is that raised awareness amongst the clinical staff about near misses in daily routines and safety barriers might have positive influence prior to checklist implementation. High checklist compliance may be challenging to maintain over time. In our hospital, adjusted team involvement was observed in a qualitative study of nurses’ experiences with the use of the Surgical Safety Checklist after one year [26]. Three strategies were identified amongst operating room nurses and nurse anaesthetists—distancing, moderating, and engaging; all influenced checklist performance and compliance [26].

### Eliminating medical errors

The high trust patients have in hospital staff mandates that we strive to achieve a zero-level of preventable incorrect surgery. Modern surgical activity could be compared to high reliability organisations (HROs). HROs are complex: They perform tasks under time pressures, with demanding activities and low incident rates or with complete absence of catastrophic failures over time [13]. However in hospitals, adverse events occur in nearly 1-in-10 in-hospital patients [1]. In HROs the system approach is primed at all levels of the organisation, with a preoccupation of the possibility of failure and with continual training on familiar scenarios [13].

A systematic review and a study of extended Surgical Time Out do not support the effectiveness of the current JC Universal Protocol in decreasing the incidence of wrong-site or wrong-level surgery [11,27]. Hence, incorrect surgery may be prevented by surface body marking of the targeted anatomical structure and by displaying these images on monitors in the operating room [11]. Additionally, intraoperative imaging by fluoroscopy and ultrasound may also confirm the diagnosis and severity in a variety of clinical situations. In cardiac surgery, trans-oesophageal echocardiography is frequently used to define the current clinical problem and may be of crucial importance in deciding the final surgical strategy.

Recently, Beuzekom and colleagues performed a controlled pre-post intervention study to minimise latent safety risk factors in the operating room and found lower perceived and reported incident rates in the intervention group [28]. In the study of Pronovost et al., eliminating medical errors and maintaining a low incident rate after implementing a checklist and infection control procedures to prevent bloodstream infections related to catheter insertions indicate acceptance of an improved standard of care [29]. Building clinical safety defences and barriers (i.e., intraoperative imaging, ultrasound and checklists) in operating rooms applies to a systematic approach for enhancing and supporting surgical team members in minimising near misses and errors [13,25].

### Systematic risk reducing efforts

To prevent near misses and incorrect surgery we suggest implementing:

•Hospital safety improvement programs

•Monitor and develop safety culture

•Chairs must report on an support safety efforts

•Build in clinical safety defences and barriers

•Surgical team training

•Perioperative checklists

### Limitations

The reports in the survey of surgical team members’ experiences of incorrect surgery are probably referring to the same occasions and cases. This could be regarded as a limitation; however, the aim of this study was not to count the numbers of actual incorrect surgeries. Since non-responder analysis was not performed, we have little information about personnel who failed to respond to our survey. Nonetheless, the response rate of 64% is quite satisfactory for an email-based survey. The questionnaire depended on surgical team members’ memory of incorrect surgery experiences at this hospital. This could have biased the accuracy of the responses. Furthermore, an observation of intraoperative routines could have added more in-depth understanding of near-miss experiences in the operating rooms.

## Conclusion

The majority of our surgical personnel experienced near misses and failures with regard to patient identity, surgical site, and/or procedure. Routines for ensuring correct patient, correct site, and correct surgical procedure were practised significantly differently by medical professionals, supporting our hypothesis. Identity check must involve all surgical team members with regard to their own medical objectives and also as a joint team responsibility. As raised awareness about near misses and inconsistent safety systems in the operating room enhances positive attitudes towards protocols, implementation of a Time Out protocol was welcomed.

We find that the study results of near-miss experiences with failure to confirm the patient identity, wrong patient being brought into the operating room, positioning of the patient on the wrong side and preparing for wrong procedures are a wake-up call for systematic risk reducing efforts and the use of checklists in surgery.

## Competing interests

The authors declare that they have no competing interests.

## Authors’ contributions

ASH contributed to data collection, carried out the analysis and drafted the manuscript. SM conceived of the study and participated in data collection, data interpretation and helped to draft the manuscript. ASH and SM contributed equally. RH and GEE contributed to interpretation of data and drafting of the manuscript. ES made substantial contributions to conceiving of the study and to drafting of the manuscript. RH, GEE and ES critically revised the manuscript for intellectual content. All authors read and approved the final manuscript.

## Authors’ information

ASH is a nurse anaesthetist, MSc and has position as PhD fellow and head nurse of the Department of Anaesthesia and Intensive Care, Haukeland University Hospital. SM work as an emergency care nurse and operating room nurse at the Department of Anaesthesia and Intensive Care, Haukeland University Hospital. RH is head of the Section of Cardiothoracic Surgery, Department of Heart Disease, Haukeland University Hospital. He is also professor at the Department of Clinical Science, Faculty of Medicine and Dentistry, University of Bergen. GEE is professor in biostatistics at the Centre for Clinical Research, Haukeland University Hospital, and at the Department of Global Public Health and Primary Health Care, Faculty of Medicine and Dentistry, University of Bergen. ES is a senior consultant anaesthesiologist, MD PhD and section leader of Head and Neck surgery at the Department of Anaesthesia and Intensive Care, Haukeland University Hospital.

## Pre-publication history

The pre-publication history for this paper can be accessed here:

http://www.biomedcentral.com/1471-2482/13/46/prepub
